# Characterization and description of *Faecalibacterium butyricigenerans* sp. nov. and *F. longum* sp. nov., isolated from human faeces

**DOI:** 10.1038/s41598-021-90786-3

**Published:** 2021-05-31

**Authors:** Yuanqiang Zou, Xiaoqian Lin, Wenbin Xue, Li Tuo, Ming-Sheng Chen, Xiao-Hui Chen, Cheng-hang Sun, Feina Li, Shao-wei Liu, Ying Dai, Karsten Kristiansen, Liang Xiao

**Affiliations:** 1 BGI-Shenzhen, Beishan Industrial Zone, Shenzhen, 518083 People’s Republic of China; 2 Laboratory of Genomics and Molecular Biomedicine, Department of Biology, University of Copenhagen, Universitetsparken 13, 2100 Copenhagen, Denmark; 3 Shenzhen Engineering Laboratory of Detection and Intervention of Human Intestinal Microbiome, BGI-Shenzhen, Shenzhen, People’s Republic of China; 4 Qingdao-Europe Advanced Institute for Life Sciences, BGI-Shenzhen, Qingdao, 266555 People’s Republic of China; 5 School of Bioscience and Biotechnology, South China University of Technology, Guangzhou, 510006 People’s Republic of China; 6 Life Sciences Institute, Zunyi Medical University, Zunyi, 563006 People’s Republic of China; 7 Institute of Medicinal Biotechnology, Chinese Academy of Medical Sciences and Peking Union Medical College, Beijing, People’s Republic of China; 8 Beijing Key Laboratory of Pediatric Respiratory Infection Diseases, Key Laboratory of Major Diseases in Children, Ministry of Education, National Clinical Research Center for Respiratory Diseases, National Key Discipline of Pediatrics (Capital Medical University), Beijing Pediatric Research Institute, Beijing Children’s Hospital, Capital Medical University, National Center for Children’s Health, Beijing, 100045 People’s Republic of China; 9 BGI College and Henan Institute of Medical and Pharmaceutical Sciences, Zhengzhou University, Zhengzhou, 450052 People’s Republic of China

**Keywords:** Bacteria, Genome, Microbiology, Genome informatics

## Abstract

Exploiting a pure culture strategy to investigate the composition of the human gut microbiota, two novel anaerobes, designated strains AF52-21^T^ and CM04-06^T^, were isolated from faeces of two healthy Chinese donors and characterized using a polyphasic approach. The two strains were observed to be gram-negative, non-motile, and rod-shaped. Both strains grew optimally at 37 °C and pH 7.0. Phylogenetic analysis based on 16S rRNA gene sequences revealed that the two strains clustered with species of the genus *Faecalibacterium* and were most closely related to *Faecalibacterium prausnitzii* ATCC 27768^T^ with sequence similarity of 97.18% and 96.87%, respectively. The two isolates shared a 16S rRNA gene sequence identity of 98.69%. Draft genome sequencing was performed for strains AF52-21^T^ and CM04-06^T^, generating genome sizes of 2.85 Mbp and 3.01 Mbp. The calculated average nucleotide identity values between the genomes of the strains AF52-21^T^ and CM04-06^T^ compared to *Faecalibacterium prausnitzii* ATCC 27768^T^ were 83.20% and 82.54%, respectively, and 90.09% when comparing AF52-21^T^ and CM04-06^T^. Both values were below the previously proposed species threshold (95–96%), supporting their recognition as novel species in the genus *Faecalibacterium*. The genomic DNA G + C contents of strains AF52-21^T^ and CM04-06^T^ calculated from genome sequences were 57.77 mol% and 57.51 mol%, respectively. Based on the phenotypic, chemotaxonomic and phylogenetic characteristics, we conclude that both strains represent two new *Faecalibacterium* species, for which the names *Faecalibacterium butyricigenerans* sp. nov. (type strain AF52-21^T^ = CGMCC 1.5206^T^ = DSM 103434^T^) and *Faecalibacterium longum* sp. nov. (type strain CM04-06^T^ = CGMCC 1.5208^T^ = DSM 103432^T^) are proposed.

## Introduction

The human gastrointestinal tract harbours complex microbial communities^1,2^, dominated by bacteria from the phyla *Bacteroidetes* and *Firmicutes*^3^. The composition and diversity of the gut microbiota are affected by numerous factors, including host genetics^4^, long-term diet^5,6^, drugs^1,7,8^, and several other environmental factors^9^. Evidence suggests that the composition of the microbiota is associated with the development of obesity^3,10–12^, diabetes^13,14^, inflammatory bowel disease^15,16^, colorectal cancer^17,18^, and non-alcoholic fatty liver disease^19,20^. Therefore, the composition and function of the microbial species living in our gut are of crucial importance for maintenance of health. Short-chain fatty acids (SCFAs) produced by fermentation of dietary fibre by several abundant genera of the intestinal microbiota, including *Roseburia*, *Eubacterium*, and *Faecalibacterium*^21^, have been reported to elicit beneficial effects on energy metabolism and prevent colonization of pathogens^22^. Bacteria of the genus *Faecalibacterium*, abundant butyric acid-producing bacteria colonizing the human gut, display anti-inflammatory effects and may be used as potential probiotics for treatment of gut inflammation^23,24^.

The genus *Faecalibacterium*, belonging to the family *Ruminococcaceae* within the order *Clostridiales*, comprises only one validated species, *Faecalibacterium prausnitzii*^25^, and two non-validated published species, ‘*Faecalibacterium moorei*’^26^ and ‘*Faecalibacterium hominis*’^27^, all originally isolated from human faeces. *F. prausnitzii* is a gram-negative, non-spore-forming, and strictly anaerobic rod-shaped bacterium. The genomic G + C content of the genus *Faecalibacterium* ranges from 47 to 57%^28^. The fermentation products from glucose are butyrate, D-lactate, and formate. In the present study, we describe two novel species of the genus *Faecalibacterium* by using a polyphasic taxonomy approach along with whole genome sequence analysis.

## Results

### Phenotypic and chemotaxonomic characterization

**Figure 1:**
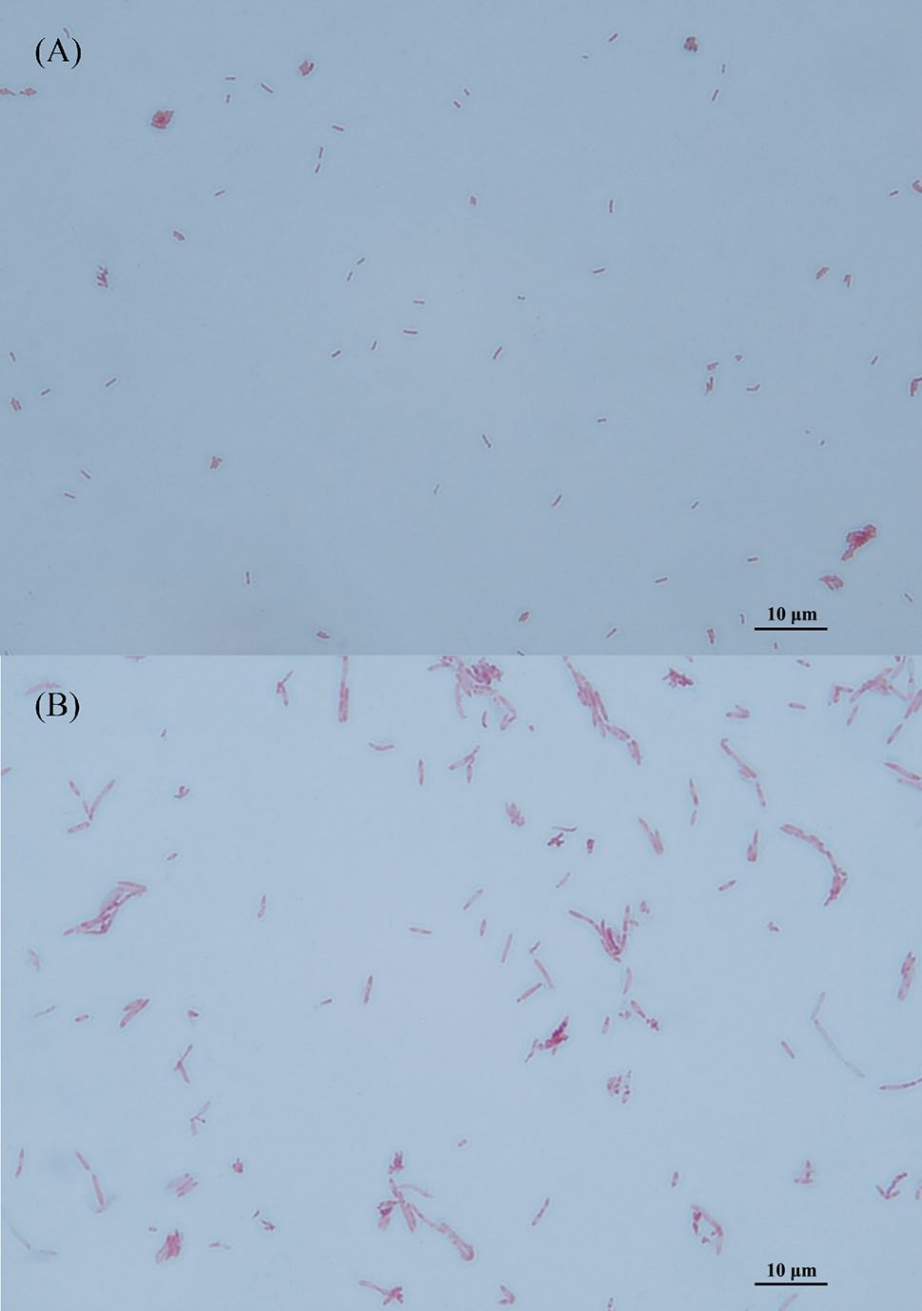
Micrographs of strains AF52-21^T^ and CM04-06^T^ after Gram staining. (**A**) AF52-21^T^; (**B**) CM04-06^T^.

**Figure 2:**
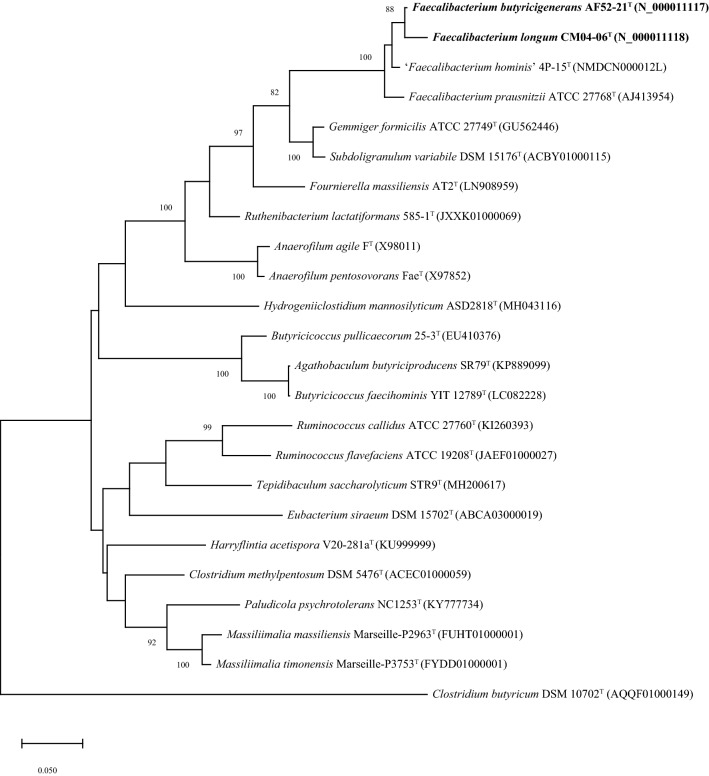
Maximum-likelihood phylogenetic tree based on 16S rRNA gene sequences showing the phylogenetic relationships of strains AF52-21^T^, CM04-06^T^ and the representatives of related taxa within the family *Ruminococcaceae*. *Clostridium butyricum* DSM 10702^T^ (AQQF01000149) was used as an out-group. Bootstrap values based on 1000 replications higher than 70% are shown at the branching points. Bar, substitutions per nucleotide position.

Strains AF52-21^T^ and CM04-06^T^ were isolated from the faeces of two healthy Chinese donors. Both strains were observed to be obligate anaerobic, gram-negative, non-spore-forming, non-motile, and rod-shaped bacteria (Fig. 1). After incubation on MPYG agar at 37 °C for 2 days, the colonies appeared 1.0–2.0 mm in diameter, round, creamy white to yellowish, convex, and opaque with entire margins for AF52-21^T^, and 2.0 mm in diameter, round, yellowish, slightly convex, and opaque with entire margins for CM04-06^T^. The growth temperature was 20–42 °C (optimum 37 °C) for AF52-21^T^ and 30–45 °C (optimum 37 °C) for CM04-06^T^. Growth was observed at pH 6.0–7.5 (optimum 7.0–7.5) for AF52-21^T^ and pH 5.0–8.0 (optimum 7.0–7.5) for CM04-06^T^. Strains AF52-21^T^ and CM04-06^T^ grew with 0–1% and 0–3% NaCl, respectively. Both strains were found to be catalase-negative. The major metabolic end products for strains AF52-21^T^ and CM04-06^T^ were acetic acid, formic acid, butyric acid, and lactic acid. Differential physiological and biochemical characteristics of strains AF52-21^T^ and CM04-06^T^ with the closest related species of genus *Faecalibacterium* are listed in the species description and in Table 1 (Fig. 2).

**Table 1:** Differential phenotypic characteristics of strains AF52-21^T^, CM04-06^T^, and the related species *F. prausnitzii* ATCC 27768^T^.

Phenotypic features	1	2	3
**Growth**			
Temperature range (optimum) (°C)	20–42 (37)	30–45 (37)	20–42 (37)
pH range	6.0–7.5	5.0–8.0	6.0–7.5
Salt tolerance (%)	1	3	3
**Fermentation products**			
formic acid (mM)	4.86	7.62	18.20
acetic acid (mM)	69.70	44.8	29.67
butyric acid (mM)	15.08	40.03	39.10
lactic acid (mM)	29.25	30.53	5.70
**Hydrolysis of**			
Aesculin	+	−	+
Gelatin	−	+	−
**Acid from (API 20A and API 50CHL)**			
Cellobiose	+	−	w
D-Fructose	w	−	+
D-Fucose	w	−	w
D-Galactose	w	−	−
D-Glucose	w	−	+
D-Lactose	+	−	−
D-Maltose	+	+	w
D-Mannitol	+	−	−
D-Mannose	+	+	−
D-Raffinose	−	w	−
D-Trehalose	+	w	w
Gluconate	−	−	+
Glycogen	+	−	−
Inositol	w	−	−
Inulin	+	−	+
Methyl-*β*-D-Xylopyranoside	w	−	−
**Enzyme activity (API ZYM)**			
*N*-acetyl-*β*-Glucosaminidase	−	w	−
Naphthol-AS-BI-Phosphohydrolase	+	−	+
*α*-Glucosidase	−	−	+
*β*-Galactosidase	−	−	w
*β*-Glucosidase	+	−	−
*β*-Glucuronidase	+	w	+
DNA G + C (mol %)	57.77	57.51	52 − 57

Strains: 1, *F. butyricigenerans* AF52-21^T^; 2, *F. longum* CM04-06^T^; 3, *F. prausnitzii* ATCC 27768^T^. + , positive; w, weakly positive; −, negative. All data are from this study.

The result of cellular fatty acid profiles of strains AF52-21^T^ and CM04-06^T^ and related species are shown in Table 2. The major components of fatty acids (constituting > 5% of the total) present in strain AF52-21^T^ were found to be C_18:1_ *ω*9*c* (39.0%), C_16:0_ (16.3%), iso-C_19:0_ (12.9%), C_18:1_ *ω*7*c* (8.1%), and C_14:0_ (5.9%). The profiles including C_18:1_ *ω*9*c* (32.5%), C_16:0_ (25.5%), iso-C_17:1_ I/anteiso B (9.7%), C_18:1_ *ω*7*c* (7.5%), and iso-C_19:0_ (5.9%) were detected as the predominant fatty acids for strain CM04-06^T^. The highest levels of fatty acids, including C_16:0_ and C_18:1_ *ω*9*c*, were found to be similar, but not identical comparing strains AF52-21^T^, CM04-06^T^, and ATCC 27768^T^. Furthermore, strains AF52-21^T^, CM04-06^T^, and ATCC 27768^T^ could be differentiated by less abundant fatty acids, such as C_18:1_ 2OH, anteiso-C_15:0_, anteiso-C_17:0_, C_13:0_ 3OH/Iso-C_15:1_ I, C_16:1_ *ω*7*c*/C_16:1_ *ω*6*c*, and antei-C_18:0_ /C_18:2_ *ω*6, 9*c* (Table 2). Strains AF52-21^T^ and CM04-06^T^ were found to contain *meso*-diaminopimelic acid as the diamino acid of the peptidoglycan. The polar lipid profiles of strains AF52-21^T^, CM04-06^T^, and *F. prausnitzii* ATCC 27768^T^ are shown in Supplementary Fig. S1. The polar lipid profiles of AF52-21^T^ and CM04-06^T^ were observed to be similar to that of the most closely related strain *F. prausnitzii* ATCC 27768^T^, with diphosphatidylglycerol (DPG), phosphatidylglycerol (PG), and several unidentified glycolipids (GL1, GL3) being present in both strains. However, the presence/absence of three unidentified lipid (L, L1, L2), unidentified phospholipid (PL), unidentified phosphoglycolipids (PGL) and an unidentified glycolipid (GL2) can be used to distinguish strains AF52-21^T^ and CM04-06^T^ from the closest relative. Quinones were not detected in either strain (Table 3).

**Table 2:** Fatty acid profiles of strains AF52-21^T^, CM04-06^T^, and the closest related species *F. prausnitzii* ATCC 27768^T^.

Fatty acids composition	*F. butyricigenerans* AF52-21^T^	*F. longum* CM04-06^T^	*F. prausnitzii* ATCC 27768^T^
C_12:0_	1.5	1.8	1.9
C_13:1_	–	–	1.25
**C**_**14:0**_	**5.9**	4.6	**11.8**
**C**_**16:0**_	**16.3**	**25.5**	**21.1**
C_17:1_ *ω*8*c*	1.3	–	1.1
**C**_**18:1**_ ***ω*7*c***	**8.1**	**7.5**	**5.7**
**C**_**18:1**_ ***ω*9*c***	**39.0**	**32.5**	**31.4**
C_18:0_	4.5	3.5	4.1
C_18:1_ 2OH	2.9	–	2.0
Iso-C_19:1_ I	1.2	1.1	2.1
**Iso-C**_**19:0**_	**12.9**	**5.9**	–
Anteiso-C_15:0_	–	2.6	–
Anteiso-C_17:0_	–	2.1	–
C_13:0_ 3OH/ Iso-C_15:1_ I	–	–	2.1
C_16:1_ *ω*7*c*/ C_16:1_ *ω*6*c*	1.5	1.9	4.0
**Iso-C**_**17:1**_** I/anteiso B**	4.7	**9.7**	**7.6**
Antei-C_18:0_ /C_18:2_ *ω*6, 9*c*	–	1.9	1.3

Numbers represent percentages of the total fatty acids. −, not detected (< 1%). All data are from this study.

**Table 3:** Levels of 16S rRNA gene sequence similarity and ANI values (in percentages) based on BLAST for strains AF52-21^T^, CM04-06^T^, and the phylogenetically related species *F. prausnitzii* ATCC 27768^T^ and the unrecognized species ‘*Faecalibacterium hominis*’ 4P-15.

Strain	Accession no.	1	2	3	4
**16S rRNA gene sequence similarity (%)**					
AF52-21^T^	N_000011117	100	98.53	97.27	98.65
CM04-06^T^	N_000011118	98.53	100	96.51	97.68
ATCC 27768^T^	AJ413954	97.27	96.51	100	98.35
4P-15	NMDCN000012L	98.65	97.68	98.35	100
**ANI values (%)**					
AF52-21^T^	CNA0017730	100	90.01	83.16	85.72
CM04-06^T^	CNA0017731	90.19	100	82.53	85.40
ATCC 27768^T^	CNA0017732	83.32	82.58	100	85.79
4P-15	NMDC60014083	85.72	85.40	85.79	100

Taxa: 1, *F. butyricigenerans* AF52-21^T^; 2, *F. longum* CM04-06^T^; 3, *F. prausnitzii* ATCC 27768^T^; 4, ‘*Faecalibacterium hominis*’ 4P-15.

### Genome analysis

**Figure 3:**
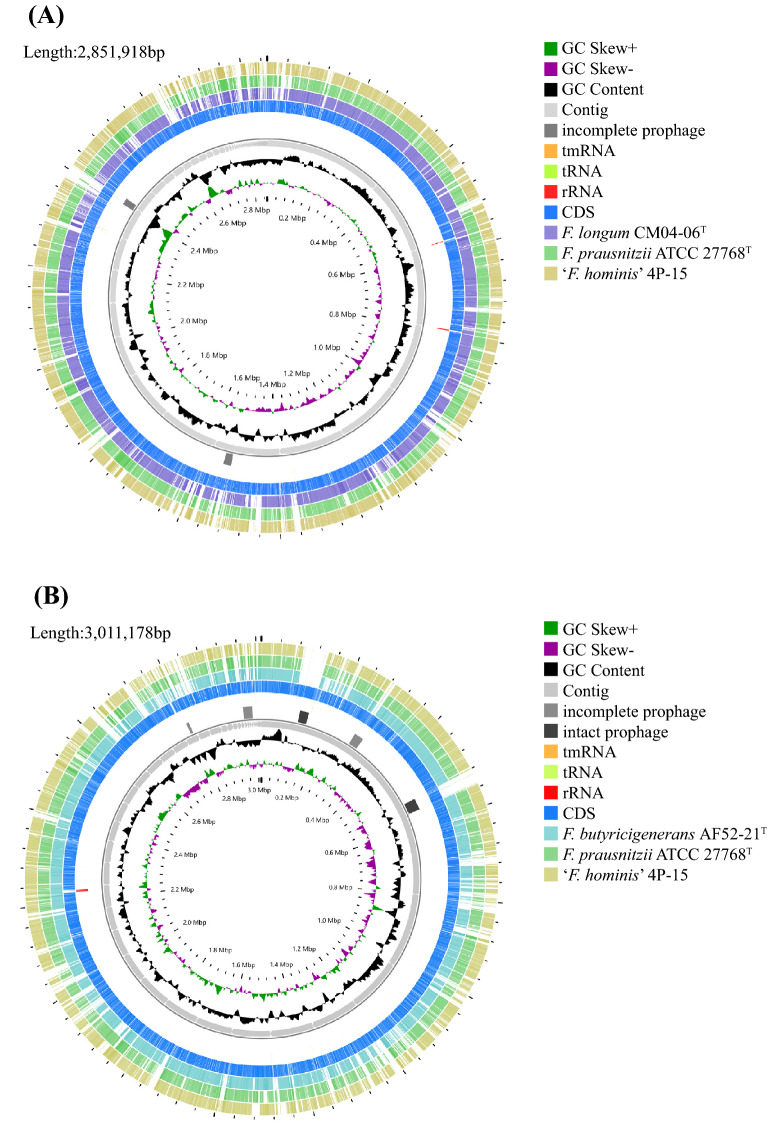
Circular map of AF52-21^T^ and CM04-06^T^. Innermost circle, GC skew; circle 2, G + C content; circle 3, contigs; circles 4, predicted prophage remnants; circle 5, tmRNA, tRNA and rRNA genes; circles 6, CDS; circles 7–9, (**A**) homologous genomic segments from CM04-06^T^, *F. prausnitzii* ATCC 27768^T^ and ‘*F. hominis*’ 4P-15, (**B**) homologous genomic segments from AF52-21^T^, *F. prausnitzii* ATCC 27768^T^ and ‘*F. hominis*’ 4P-15.

The assembled draft genomes of strains AF52-21^T^ and CM04-06^T^ comprised total lengths of 2,851,918 bp and 3,011,178 bp with 73 and 47 scaffolds, respectively (Table 4). The G + C contents calculated from the genome sequences were 57.77% and 57.51%, which are slightly higher than the range reported previously for the genus *Faecalibacterium* (47–57 mol%)^25^. CheckM analysis of the genomes showed high completeness (> 90%) and low contamination (< 5%) (Table 4), indicating these are high-quality genomes sequences. The genome comparison between strains AF52-21^T^, CM04-06^T^, ATCC 27768^T^, and ‘*Faecalibacterium hominis*’ 4P-15 showed ANI values ranging from 82.53% to 90.19% (Table 3), which are significantly below the proposed cutoff value of 95–96% for delineating bacterial species, indicating that strains AF52-21^T^ and CM04-06^T^ represent novel species in the genus *Faecalibacterium*. Circular maps of the two strains AF52-21^T^ and CM04-06^T^ are shown in Fig. 3.

**Table 4:** Genome properties of *F. butyricigenerans* AF52-21^T^ and *F. longum* CM04-06^T^.

Feature	AF52-21^T^	CM04-06^T^
Accession no.	CNA0017730	CNA0017731
Approximate genome Size (bp)	2,851,918	3,011,178
G + C content (mol%)	57.77	57.51
DNA scaffolds	73	47
N50 Length	191,233	119,299
Completeness	100	99.32
Contamination	0	0
Genes total number	2291	2506
Gene average length (bp)	939	920
rRNAs (5S, 16S, 23S)	4	5
tRNAs	60	61
sRNA	0	0
Genes assigned to COGs	2029	2164

### 16S rRNA gene sequence extraction and phylogenetic analysis

The almost complete 16S rRNA gene sequences of strains AF52-21^T^ and CM04-06^T^ were extracted from the genomes, in which the locations are Scaf2_220520-222018 and Scaf13_51882-53380, respectively. The length of 16S rRNA gene sequences was found to be 1499 bp for both strains. BLAST analysis of the 16S rRNA gene sequences against the EzBioCloud server showed that the two strains are most closely related to *F. prausnitzii* ATCC 27768^T^, which is the sole valid species of the genus *Faecalibacterium*, with similarity values of 97.27% and 96.51%, respectively. Strains AF52-21^T^ and CM04-06^T^ share a 16S rRNA gene sequence similarity of 98.65% and 97.68% with ‘*Faecalibacterium hominis*’ 4P-15. The 16S rRNA gene sequence similarity between strains AF52-21^T^ and CM04-06^T^ is 98.53% (Table 3). All these values are lower than the recommended threshold (98.7%) for classification of human-associated bacterial isolates at the species level^29^. Phylogenetic analysis based on the maximum-likelihood, neighbour-joining, and minimum-evolution (Fig. 2, Supplementary Figs. S2 and S3, respectively) confirmed the affiliation of the novel isolates with the genus *Faecalibacterium*, revealing that the two isolates form a distinct cluster with *F. prausnitzii* ATCC 27768^T^, supported independently of the treeing method by a high bootstrap value*.*

### Function annotation

For genome annotation, the distributions of the genes into clusters of orthologous groups (COGs) functional categories are depicted in Supplementary Fig. S4 and Table S1. Both strains AF52-21^T^ and CM04-06^T^ share identical COGs functional categories, but different functional genes numbers. Annotated genes associated with synthesis of diaminopimelic acid (DAP), teichoic and lipoteichoic acids, lipopolysaccharides, and metabolism of polar lipids and polyamines by RAST annotation, comparing strains AF52-21^T^ and CM04-06^T^ with ATCC 27768^T^ are shown in Table S2. For strain AF52-21^T^, 11 genes/proteins were observed to be associated with biosynthesis of DAP, 18 genes/proteins with biosynthesis of polar lipids, 12 genes/proteins with biosynthesis of polyamines, 3 genes/proteins with biosynthesis of teichoic and lipoteichoic acids, and 14 genes/proteins with biosynthesis of quinones. For strain CM04-06^T^, 12 genes/proteins were found to be associated with biosynthesis of DAP, 19 genes/proteins with biosynthesis of polar lipids, 13 genes/proteins with biosynthesis of polyamines, 2 genes/proteins with biosynthesis of teichoic and lipoteichoic acids, and 16 genes/proteins with biosynthesis of quinones. We detected no genes involved in the biosynthesis of lipopolysaccharides or mycolic acids in strains AF52-21^T^ and CM04-06^T^.

**Figure 4:**
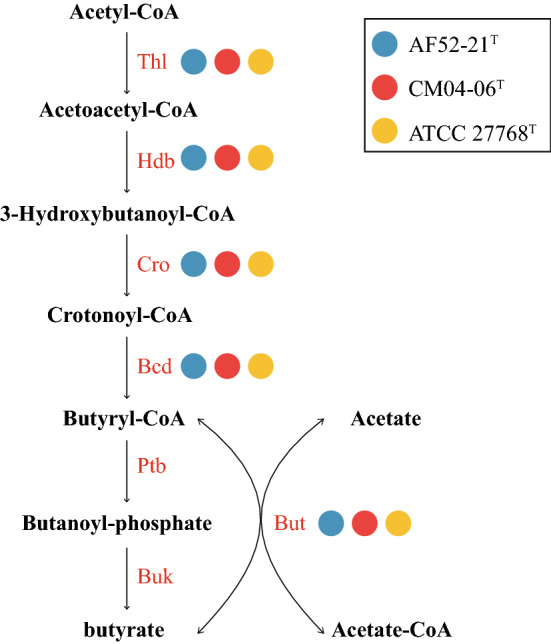
The synthesis pathways from acetyl-CoA to butyrate. Strains AF52-21^T^, CM04-06^T^ and ATCC 27768^T^ are presented as blue, red, and yellow, respectively. *Thl*, thiolase; *Hdb*, *β*-hydroxybutyryl-CoA dehydrogenase; *Cro*, crotonase; *Bcd*, butyryl-CoA dehydrogenase; *But*, butyryl-CoA:acetate CoA transferase; *Ptb*, phosphate butyryltransferase; *Buk*, butyrate kinase.

**Figure 5:**
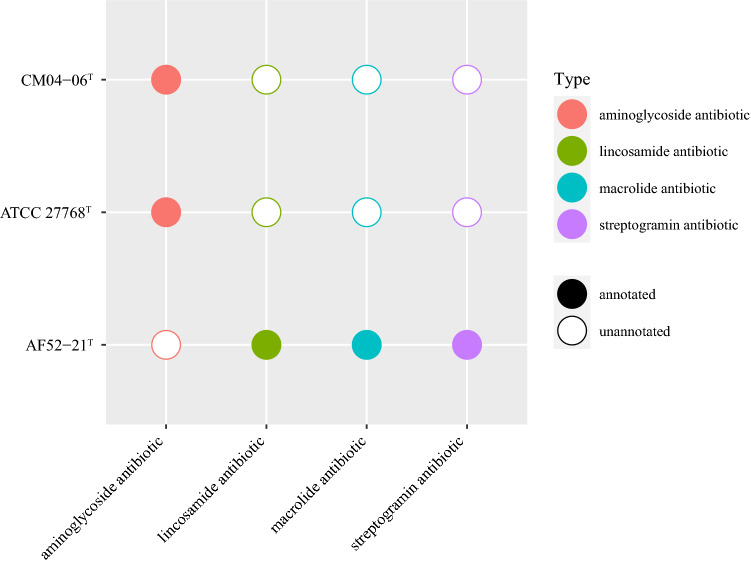
Comparison of antibiotics genes in strains AF52-21^T^, CM04-06^T^, and *F. prausnitzii* ATCC 27768^T^.

The functional annotation showed that AF52-21^T^, CM04-06^T^, and ATCC 27768^T^ contain a complete acetyl-CoA to butyrate synthesis pathway, but possess butyryl-CoA:acetate CoA-transferase activity only in the final step (Fig. 4), as discussed previously^30,31^. The antiSMASH analysis of biosynthetic gene clusters (BGCs) showed that strains AF52-21^T^ and CM04-06^T^ both contain two potential BGCs, which encode bacteriocin and sactipeptide, respectively, while ATCC 27768^T^ contains BGCs encoding microcin and sactipeptide, respectively (Supplementary Fig. S5). Prophages were identified using the PHAST software, and the results are shown in Supplementary Fig. S6. Two incomplete phage sequences were detected in the AF52-21^T^ genome, one of which encodes the Phd_YefM protein, an antitoxin component. Three incomplete phage sequences and two intact prophages were detected in the CM04-06^T^ genome, encoding the Phd_YefM protein, relaxase/mobilisation nuclease domain, bacterial mobilisation protein (MobC) /ribbon-helix-helix protein, helix-turn-helix, and predicted transcriptional regulators. Moreover, the antibiotic resistance analysis indicated that strain AF52-21^T^ contains macrolide antibiotic, lincosamide antibiotic, and streptogramin antibiotic genes, while strains CM04-06^T^ and ATCC 27768^T^ contain aminoglycoside antibiotic genes (Fig. 5). To better understand the biosynthetic pathway contributing to the in vitro characteristics of strains AF52-21^T^ and CM04-06^T^, we explored genes related to important pathways involved in carbohydrate metabolism. The comparison of in vitro and in silico characteristics is presented in Table 5.

**Table 5:** Comparison of in vitro and in silico characteristics.

Metabolic substrate or product	AF52-21^T^		CM04-06^T^		ATCC 27768^T^	
	In silico	In vitro	In silico	In vitro	In silico	In vitro
Cellobiose	GH2, GH3	+	GH1, GH2, GH3, GH88	–	GH1, GH2, GH3, GH4, GH88, GH94	w
Galactose	GH2	w	GH2	–	GH2, GH36	–
Glucose	GH31	w	GH1, GH31	–	GH1, GH31, GH33, GH43	+
Lactose	GH2, GH31	+	GH1, GH2, GH31	–	GH1, GH2, GH31, GH36, GH43	–
Maltose	GH13, GH13_20, GH13_39, GH77, GT35	+	GH1, GH13, GH13_20, GH13_39, GH77, GT35	+	GH1, GH13, GH13_20, GH13_39, GH77, GT35	w
Mannose	GH2, GH3	+	GH2, GH3	+	GH2, GH3	–
Raffinose	GH2, GH13, GH32	–	GH2, GH13, GH32	w	GH2, GH4, GH13, GH32, GH36	–
Trehalose	GH13	+	GH13	w	GH13	w
Glycogen	GH13, GH13_9, GH13_39, GT5, GT35	+	GH13, GH13_9, GH13_39, GT5, GT35	–	GH13, GH13_9, GH13_39, GT5, GT35	–
Inulin	GH32	+	GH32	–	GH32	+
butyric acid	butyryl-CoA:acetate CoA transferase	+	butyryl-CoA:acetate CoA transferase	+	butyryl-CoA:acetate CoA transferase	+

All data are from this study.

*GH* glycoside hydrolases, *GT* glycosyl transferases, + , positive, *w* weakly positive; − , negative.

## Discussion

16S rRNA gene phylogeny, genome sequence comparison, and physiological results showed that the two new isolates AF52-21^T^ and CM04-06^T^ represent two novel species. The ANI values between AF52-21^T^, CM04-06^T^ and the closest related species ATCC 27768^T^ were found to be 82.54% and 90.09%, respectively, which support the delineation of new species. The result of biochemical and genomic functional analyses showed that both strains AF52-21^T^ and CM04-06^T^ are butyric acid-producing bacteria.

Most strains in the genus *Faecalibacterium* exhibit a common ability to produce butyric acid, bioactive peptides, and other anti-inflammatory substances with immunomodulatory effects^23,24,32^. Several studies have confirmed that a decreased abundance of this genus is related to the occurrence and development of inflammatory bowel diseases^33–35^. Accordingly, bacteria of the genus *Faecalibacterium* are receiving much attention as possible candidate next-generation probiotics (NGPs), which may be used for disease treatment^36,37^.

Previous studies based on comparative genomics of isolates suggested a wide diversity of this genus, with the presence of at least two phylotypes in *F. prausnitzii*^26^. A recent study analysing the *Faecalibacterium*-like MAGs, proposed that *Faecalibacterium* from the human gut can be divided into 12 clades^37^. These studies have expanded the diversity of the genus *Faecalibacterium* and proposed that different phylotypes have different functions with potentially different contributions in relation to health or diseases.

Moreover, as a candidate taxa for the NGPs, the bacteria of the genus *Faecalibacterium* can be used for in vitro functional verification and animal model experiments to further explore possible probiotic functions, and ultimately, used in clinical disease intervention trials.

### Emended description the genus of *Faecalibacterium*

The genus description is as given by Duncan *et al*^25^ with the following changes. Cells are able to produce formic acid, acetic acid, and butyric acid. The major polar lipids are diphosphatidylglycerol, phosphatidylglycerol and several unidentified glycolipids. Genomic DNA G + C content is 47–63 mol%. Genome size is 2.68–3.32 Mb.

### Emended description of *Faecalibacterium prausnitzii*

Cells are able to produce formic acid, acetic acid, butyric acid, and lactic acid. The major fatty acids (constituting > 5% of the total) are C_16:0_, C_18:1_ *ω*7*c*, and C_18:1_ *ω*9*c*. The rest of the species characteristics are as described by Cato *et al*^38^, Duncan *et al*^25^, and Fitzgerald *et al*^26^. The type strain is *Faecalibacterium prausnitzii* ATCC 27768^T^ (= NCIMB 13872^T^).

### Description of *Faecalibacterium butyricigenerans* sp. nov.

*Faecalibacterium butyricigenerans* (bu.ty.ri.ci.ge′ne.rans. N.L. n. *acidum butyricum* butyric acid; L. part. adj. *generans*, producing; N.L. adj. *butyricigenerans*, butyric acid-producing; referring to its production of butyric acid).

Cells are gram-negative, non-motile, non-spore-forming and rod-shaped. Strictly anaerobic and catalase negative. Colonies on PYG agar are round, creamy white to yellowish, convex, and opaque with entire margins, and colony size is approximately 1.0–2.0 mm in diameter after incubation at 37 °C for 2 days. Cells are able to grow at 20–42 °C with optimum temperature at 37 °C. The pH range for growth is 6.0–7.5 (optimum at 7.0–7.5). Growth occurs at NaCl concentrations 0–1%. Indole is not produced. Positive for hydrolysis of esculin and negative for gelatin. Formic acid, acetic acid, butyric acid, and lactic acid are the fermentation products. The major fatty acids are C_14:0_, C_16:0_, C_18:1_ *ω*7*c*, C_18:1_ *ω*9*c*, and iso-C_19:0_.

The type strain, AF52-21^T^ (= CGMCC 1.5206^T^ = DSM 103434^T^), was isolated from human faeces. The G + C content of the genomic DNA is 57.77 mol% as calculated from whole genome sequencing.

### Description of *Faecalibacterium longum* sp. nov.

*Faecalibacterium longum* (lon′gum. L. neut. adj. *longum* long, the shape of the cells).

Cells are gram-negative, non-motile, non-spore forming, long rod in shape. Strictly anaerobic. Catalase and urease are negative. Colonies are round, yellowish, slightly convex, and opaque with entire margins with 2.0 mm in diameter on PYG agar for incubation at 37 °C for 48 h under anaerobic condition. The strain shows growth at 30–45 °C (optimum temperature is 37 °C). Growth is observed at pH 5.0–8.0 (optimum pH is 7.0–7.5). NaCl is tolerated with concentrations up to 3%. Indole is not produced. Gelatin is hydrolysed, but aesculin is not. Major end products are acetic acid, formic acid, butyric acid, and lactic acid. The major fatty acids (constituting > 5% of the total) are C_16:0_, C_18:1_ *ω*7*c*, C_18:1_ *ω*9*c*, iso-C_19:0_, and iso-C_17:1_ I/anteiso B.

The type strain, CM04-06^T^ (= CGMCC 1.5208^T^ = DSM 103432^T^), was isolated from human faeces. The G + C content of the genomic DNA is 57.51 mol% as calculated from whole genome sequencing.

## Methods

### Origin of bacterial strains

Faeces samples were collected from two healthy donors living in Shenzhen, Guangdong province, China, one donor is an adult female (AF), and the other is a male child (CM). The samples were stored refrigerated and kept anaerobically until processed. The collection of the samples was approved by the Institutional Review Board on Bioethics and Biosafety of BGI under number BGI-IRB17005-T2. All protocols were in compliance with the Declaration of Helsinki and explicit informed consent was obtained from the participant and the parents of the male child. 1 g of faecal sample was diluted with 0.1 M PBS (pH 7, supplemented with 0.5% cysteine) and spread onto modified peptone-yeast extract-glucose (MPYG, supplemented with 5 g/L sodium acetate in DSMZ 104 medium) agar plates in an anaerobic box (Bactron Anaerobic Chamber, BactronIV-2, shellab, USA). The plates were incubated at 37 °C under anaerobic conditions (90% N_2_, 5% CO_2_, and 5% H_2_, v/v) for 3–5 days. Single colonies were randomly picked and purified by repetitive subculturing on the new plates containing the same medium and incubated under the same conditions as described above. Among the pure cultures, two isolates, designated as AF52-21^T^ and CM04-06^T^, respectively, were obtained and subsequently maintained in 20% (v/v) glycerol and frozen at -80 °C.

### Phenotypic characterization

The morphological characteristics of strains AF52-21^T^ and CM04-06^T^ were performed on cultures grown on MPYG medium at 37 °C. Bacterial cell shape was examined by phase contrast microscopy (Olympus BX51, Japan) during the exponential phase of growth. Cell motility was examined using semi-solid MPYG medium containing 0.5% agar^39^. The Gram reaction was carried out using a Gram-staining kit (Solarbio, China). Spore formation and presence of flagella were determined by staining using spore stain kit and flagella stain kit supplied by Solarbio (China) following the manufacturer’s instructions. Colony morphology was observed following growth of the cultures on PYG agar for 2 days at 37 °C. Optimal temperature for growth was determined using growth in MPYG medium at 4, 10, 20, 25, 30, 35, 37, 45, and 50 °C for 7 days. The pH range for growth was also measured in MPYG medium covering the range of pH 3.0–10.0 (at an interval of 0.5 pH units) at 37 °C for 7 days, and the pH test medium stabilized with the appropriate buffers as described by Sorokin^40^. Growth at various NaCl concentrations (0–6%, in increments of 1.0%) was performed for determining tolerance to NaCl. Catalase activity was assessed by gas formation after dropping the fresh cells in 3% H_2_O_2_ solution. Biochemical properties, including utilization of substrates, acid production from carbohydrates, enzyme activities, hydrolytic activities, were determined using the API 20A, API 50CHL, and API ZYM systems (bioMérieux Inc., Marcy-l’Étoile, France) according to the manufacturer’s instructions with modification by adding sodium acetate at concentration of 0.5% in all tests. The reference type strain was tested under the same condition as used for strains AF52-21^T^ and CM04-06^T^. In all tests, the strains were incubated under anaerobic conditions.

### Chemotaxonomic characteristics

Chemotaxonomic features were investigated by analysing of cellular fatty acids, cell wall composition, polar lipids, and quinones. Biomasses of strains AF52-21^T^, CM04-06^T^, and ATCC 27768^T^ were harvested from cells growing in MPYG at 37 °C under anaerobic conditions for 2 days. Whole cell fatty acid methyl esters (FAMEs) were extracted, separated and identified according to the MIDI Microbial Identifications System and performed by CGMGG (China General Microbiological Culture Collection Center, Beijing, China) identification service. The diagnostic isomer of diaminopimelic acid in whole-cell hydrolysates was identified by TLC as described by Zou et al.^41^. The polar lipids of strain AF52-21^T^, CM04-06^T^, and ATCC 27768^T^ were extracted from lyophilized bacterial cells and analysed using two-dimensional TLC as described^42^. Menaquinone components were extracted and identified by HPLC (LC-20AD; Shimadzu) coupled with a single quadrupole mass spectrometer (LCMS-2020; Shimadzu) as described^42^.

### Fermentation products analysis

For analysis the metabolic end products from glucose fermentation, including SCFAs and organic acids, cells were cultured in MPYG broth at 37 °C under anaerobic conditions for 2 days. Supernatant harvested from the cultures centrifuged at 10,000 g for 10 min was used for determining SCFAs and organic acids. SCFAs detection was performed using a gas chromatograph (GC-7890B, Agilent) equipped with a flame ionization detector (FID) and capillary column packed with Agilent 19091 N-133HP-INNOWax porapak HP-INNOWax (30 m × 0.25 mm × 0.25 μm). Organic acids were analysed by equipping capillary column packed with Agilent 122-5532G DB-5 ms (40 m × 0.25 mm × 0.25 μm).

### Genome sequencing, assembly, and annotation of isolates

For genome sequences of strains AF52-21^T^ and CM04-06^T^, genomic DNA was extracted following the method described above. The draft genome was sequenced on an Ion Proton Technology (Life Technologies) platform at BGI-Shenzhen (Shenzhen, China) after constructing a paired-end DNA library with insert size of 500 bp. The resulting reads were assembled using the SOAPdenovo 2 package^43^. CheckM (v1.1.2) was used to estimate genome completeness and contamination^44^. Genome assemblies were visualized using CGView Server^45^ (http://stothard.afns.ualberta.ca/cgview_server/index.html). Annotation of the assembled genome was performed using the Rapid Annotation Using Subsystem Technology (RAST) server^46^ and COG database^47^. The G + C content in genomic DNA was calculated from the whole genome sequence. The genes in known pathways from acetyl-CoA to butyrate were annotated by BLAST (evalue = 1e−5, identity ≥ 60%, coverage ≥ 90%)^30^. AntiSMASH 5.0 was used to predict BGCs. A search for prophages was performed by PHAST (http://phast.wishartlab.com/)^48^. Antibiotic resistance was analysed using the CARD database^49^. The carbohydrate active enzymes genes were annotated by dbCAN2^50^. The dbCAN-PUL^51^ database was used to determine genes related to important carbohydrate metabolism pathways.

### Average nucleotide identities

Genome relatedness was investigated by calculating average nucleotide identity (ANI)^52^, with a value of 95–96% proposed for delineating bacterial species, corresponding to the traditional 70% DNA-DNA reassociation standard^53,54^. The ANI values between strains AF52-21^T^, CM04-06^T^, and closely related species were determined using the FastANI^55^.

### Phylogenetic analysis based on 16S rRNA genes sequence

16S rRNA gene sequences were extracted from the genomes using RNAmmer^56^. The obtained 16S rRNA gene sequences of strains AF52-21^T^ and CM04-06^T^ were compared with the sequences of type strains retrieved from the EzBioCloud database (https://www.ezbiocloud.net/)^57^ and an unrecognized species ‘*Faecalibacterium hominis*’ 4P-15^27^ using the BLAST program to determine the nearest phylogenetic neighbours and 16S rRNA gene sequence similarity values. Phylogenetic trees were reconstructed by using the neighbour-joining method^58^ (K2 + G model of substitution), maximum-likelihood method^59^ (GTR + G + I model of substitution) and minimum-evolution method^60^ (K2 + G model of substitution) with the MEGA X program package^61^, after Clustal W multiple alignment of the sequences. 1548 nucleotide positions were finally used for tree constructions. Robustness of the phylogenetic trees was evaluated by using the bootstrap resampling method (1000 resamplings) of Felsenstein^62^.

## Supplementary Information

Supplementary Information.

## Data Availability

The China National GeneBank DataBase (CNGBdb)^63^ accession numbers for the 16S rRNA gene sequences determined in this study are: AF52-21^T^ (N_000011117) and CM04-06^T^ (N_000011118). The data of draft genome sequences have been deposited into CNGB Sequence Archive (CNSA)^64^ of CNGBdb with accession number CNA0017730 and CNA0017731 for strains AF52-21^T^ and CM04-06^T^, respectively.
