# Supplementary material for: Characterization and description of Faecalibacterium butyricigenerans sp. nov. and F. longum sp. nov., isolated from human faeces
Source: Sci Rep. 2021 May 31;11:11340. doi: 10.1038/s41598-021-90786-3 (PMC8166934; doi:10.1038/s41598-021-90786-3)

Supplementary Material

Characterization and description of *Faecalibacterium butyricigenerans* sp. nov. and *F. longum* sp. nov., isolated from human faeces

Yuanqiang Zou[1, 2, 3, 4†*], Xiaoqian Lin[1, 5†], Wenbin Xue[1], Li Tuo[6], Ming-Sheng Chen[6], Xiao-Hui Chen[6], Cheng-hang Sun[7], Feina Li[8], Shao-wei Liu[7], Ying Dai[1], Karsten Kristiansen[1, 2, 4], Liang Xiao[1, 3, 4, 9*]

[1] BGI-Shenzhen, Shenzhen 518083, China

[2] Laboratory of Genomics and Molecular Biomedicine, Department of Biology, University of Copenhagen, Universitetsparken 13, 2100 Copenhagen, Denmark

[3] Shenzhen Engineering Laboratory of Detection and Intervention of human intestinal microbiome, BGI-Shenzhen, Shenzhen, China

[4] Qingdao-Europe Advanced Institute for Life Sciences, BGI-Shenzhen, Qingdao 266555, China

[5] School of Bioscience and Biotechnology, South China University of Technology, Guangzhou 510006, China

[6] Life Sciences Institute, Zunyi Medical University, Zunyi 563006, P. R. China

[7] Institute of Medicinal Biotechnology, Chinese Academy of Medical Sciences and Peking Union Medical College, Beijing, China

[8] Beijing Key Laboratory of Pediatric Respiratory Infection Diseases, Key Laboratory of Major Diseases in Children, Ministry of Education, National Clinical Research Center for Respiratory Diseases, National Key Discipline of Pediatrics (Capital Medical University), Beijing Pediatric

Research Institute, Beijing Children's Hospital, Capital Medical University, National Center for Children's Health, Beijing 100045, China

[9] BGI College & Henan Institute of Medical and Pharmaceutical Sciences, Zhengzhou University, Zhengzhou 450052, China

Supplementary Table S1. Number of genes associated with general COG functional categories in the genome of *F. butyricigenerans* AF52-21^T and *F. longum* CM04-06^T.

function code	function	AF52-21^T		CM04-06^T		ATCC 27768^T	
		Gene Count	% of Total Genes	Gene Count	% of Total Genes	Gene Count	% of Total Genes
C	Energy production and conversion	100	4.37%	99	3.95%	103	4.13%
D	Cell cycle control, cell division, chromosome partitioning	34	1.49%	43	1.72%	47	1.89%
E	Amino acid transport and metabolism	184	8.04%	186	7.42%	173	6.94%
F	Nucleotide transport and metabolism	74	3.23%	74	2.95%	72	2.89%
G	Carbohydrate transport and metabolism	176	7.69%	181	7.22%	209	8.39%
H	Coenzyme transport and metabolism	91	3.98%	98	3.91%	92	3.69%
I	Lipid transport and metabolism	64	2.80%	68	2.71%	64	2.57%
J	Translation, ribosomal structure and biogenesis	200	8.74%	200	7.98%	201	8.07%
K	Transcription	155	6.77%	185	7.38%	196	7.87%
L	Replication, recombination and repair	136	5.94%	153	6.11%	156	6.26%
M	Cell wall/membrane/envelope biogenesis	114	4.98%	111	4.43%	116	4.65%
N	Cell motility	12	0.52%	8	0.32%	14	0.56%
O	Posttranslational modification, protein turnover, chaperones	74	3.23%	78	3.11%	79	3.17%
P	Inorganic ion transport and metabolism	92	4.02%	101	4.03%	75	3.01%
Q	Secondary metabolites biosynthesis, transport and catabolism	18	0.79%	20	0.80%	19	0.76%
R	General function prediction only	170	7.43%	178	7.10%	176	7.06%
S	Function unknown	89	3.89%	93	3.71%	98	3.93%

T	Signal transduction mechanisms	98	4.28%	105	4.19%	109	4.37%
U	Intracellular trafficking, secretion, and vesicular transport	32	1.40%	35	1.40%	38	1.52%
V	Defense mechanisms	78	3.41%	100	3.99%	77	3.09%
X	Mobilome: prophages, transposons	37	1.62%	47	1.88%	36	1.44%
Z	Cytoskeleton	1	0.04%	1	0.04%	2	0.08%
Not in COG	Not in COG	260	11.36%	342	13.65%	340	13.64%

Supplementary Table S2. The specific genes/protein related to biosynthesis of DAP, polar lipids, polyamines and lipoteichoic and teichoic acids and their positions in the genome in comparation of strains AF52-21^{T}, CM04-06^{T} and related organism, ATCC 27768^{T} identified by Rapid Annotation Subsystem Technology (RAST). (A) DAP. (B) Polar lipids. (C) Polyamines. (D) Teichoic and lipoteichoic acids. (E) Quinones.

A.

Gene/Protein related to DAP	AF52-21^{T}	CM04-06^{T}	ATCC 27768^{T}
4-hydroxy-tetrahydrodipicolinate reductase (EC 1.17.1.8)	AF52-21.Scaf1: 248922..249680	CM04-06A.Scaf1: 485465..484707	ATCC-27768.Scaf2: 112025..112783
4-hydroxy-tetrahydrodipicolinate synthase (EC 4.3.3.7)	AF52-21.Scaf1: 247952..248845	CM04-06A.Scaf1: 486434..485541	ATCC-27768.Scaf2: 111058..111951
		CM04-06A.Scaf1: 708125..707226	ATCC-27768.Scaf8: 9055..9969
Aspartate-semialdehyde dehydrogenase (EC 1.2.1.11)	AF52-21.Scaf1: 246824..247906	CM04-06A.Scaf1: 487595..486483	ATCC-27768.Scaf2: 109886..111013
Aspartokinase (EC 2.7.2.4)	AF52-21.Scaf1: 251066..251533	CM04-06A.Scaf1: 315482..316681	ATCC-27768.Scaf2: 114173..114640
	AF52-21.Scaf4: 41612..40413	CM04-06A.Scaf1: 483321..482854	ATCC-27768.Scaf4: 189132..187933
Diaminopimelate decarboxylase (EC 4.1.1.20)	AF52-21.Scaf1: 151844..153148	CM04-06A.Scaf1: 586988..585690	ATCC-27768.Scaf4: 145766..144480
Diaminopimelate epimerase (EC 5.1.1.7)	AF52-21.Scaf6: 41663..42502	CM04-06A.Scaf6: 50667..49828	ATCC-27768.Scaf11: 13597..12758
L,L-diaminopimelate aminotransferase (EC 2.6.1.83)	AF52-21.Scaf6: 42572..43759	CM04-06A.Scaf6: 49718..48531	ATCC-27768.Scaf11: 12643..11456
N-acetyl-L,L-diaminopimelate deacetylase (EC 3.5.1.47)	AF52-21.Scaf3: 214671..213451	CM04-06A.Scaf6: 3512..4744	ATCC-27768.Scaf13: 47182..45965
UDP-N-acetylmuramoylalanyl-D-glutamate--2,6-diaminopimelate ligase (EC 6.3.2.13)	AF52-21.Scaf2: 145885..147363	CM04-06A.Scaf16: 38892..37414	ATCC-27768.Scaf6: 58143..59621
UDP-N-acetylmuramoylalanyl-D-glutamyl-2,6-diaminopimelate--D-alanyl-D-alanine ligase (EC 6.3.2.10)	AF52-21.Scaf2: 139527..140900	CM04-06A.Scaf16: 45305..43932	ATCC-27768.Scaf6: 51597..52979

B.

Gene/Protein related to Polar lipids	AF52-21^T	CM04-06^T	ATCC 27768^T
1-acyl-sn-glycerol-3-phosphate acyltransferase (EC 2.3.1.51)	AF52-21.Scaf5: 64236..63628	CM04-06A.Scaf2: 103211..102486	ATCC-27768.Scaf1: 34950..35678
	AF52-21.Scaf8: 134315..135040	CM04-06A.Scaf5: 66767..67375	ATCC-27768.Scaf3: 75990..75379
ABC-type multidrug/protein/lipid transport system, ATPase component	AF52-21.Scaf7: 144246..145862	CM04-06A.Scaf1: 751898..753583	ATCC-27768.Scaf4: 202508..204319
Acyl carrier protein	AF52-21.Scaf2: 74763..75008	CM04-06A.Scaf19: 16014..16259	ATCC-27768.Scaf11: 13875..14111
	AF52-21.Scaf6: 41453..41019	CM04-06A.Scaf6: 50968..51201	ATCC-27768.Scaf3: 222636..222394
	AF52-21.Scaf6: 147592..147359	CM04-06A.Scaf8: 71225..71458	ATCC-27768.Scaf6: 59865..60092
Acyl-phosphate:glycerol-3-phosphate O-acyltransferase PlsY	AF52-21.Scaf3: 55591..54929	CM04-06A.Scaf1: 40829..41491	ATCC-27768.Scaf7: 80759..81421
Alcohol dehydrogenase (EC 1.1.1.1)			ATCC-27768.Scaf8: 7868..9034
Alcohol dehydrogenase (EC 1.1.1.1); Acetaldehyde dehydrogenase (EC 1.2.1.10)			ATCC-27768.Scaf3: 236571..235354
Cardiolipin synthetase (EC 2.7.8.-)	AF52-21.Scaf17: 953..2512	CM04-06A.Scaf1: 238636..240174	ATCC-27768.Scaf12: 86311..87870
	AF52-21.Scaf4: 118625..117087	CM04-06A.Scaf11: 75601..77217	ATCC-27768.Scaf21: 33225..34841
	AF52-21.Scaf9: 107406..109022		ATCC-27768.Scaf5: 85467..87008
CDP-diacylglycerol--glycerol-3-phosphate 3-phosphatidyltransferase (EC 2.7.8.5)	AF52-21.Scaf18: 1492..2073	CM04-06A.Scaf14: 31899..32480	ATCC-27768.Scaf10: 61777..61178
Dihydroxyacetone kinase family protein	AF52-21.Scaf4: 53472..55166	CM04-06A.Scaf1: 303303..301609	ATCC-27768.Scaf4: 34861..36555
Glycerate kinase (EC 2.7.1.31)		CM04-06A.Scaf3: 103434..103234	ATCC-27768.Scaf11: 29557..29357
Glycerol kinase (EC 2.7.1.30)	AF52-21.Scaf4: 162121..160622	CM04-06A.Scaf1: 725198..723699	ATCC-27768.Scaf6: 5227..3725

Glycerol-3-phosphate dehydrogenase (EC 1.1.5.3)			ATCC-27768.Scaf13: 45121..43676
Glycerol-3-phosphate dehydrogenase [NAD(P)+] (EC 1.1.1.94)	AF52-21.Scaf11: 76245..77270	CM04-06A.Scaf21: 25439..24414	ATCC-27768.Scaf7: 41466..42500
Octaprenyl diphosphate synthase (EC 2.5.1.90) / Dimethylallyltransferase (EC 2.5.1.1) / (2E,6E)-farnesyl diphosphate synthase (EC 2.5.1.10) / Geranylgeranyl pyrophosphate synthetase (EC 2.5.1.29)		CM04-06A.Scaf1: 473857..474747	
Membrane-associated phospholipid phosphatase			ATCC-27768.Scaf3: 172565..173101
Phosphate:acyl-ACP acyltransferase PlsX	AF52-21.Scaf3: 103602..102595	CM04-06A.Scaf3: 7444..6437	ATCC-27768.Scaf12: 49665..50681
Phosphatidate cytidylyltransferase (EC 2.7.7.41)	AF52-21.Scaf4: 65101..64241	CM04-06A.Scaf1: 292927..293787	ATCC-27768.Scaf4: 45295..44435
Phosphatidylglycerophosphatase B (EC 3.1.3.27)	AF52-21.Scaf11: 94463..93924	CM04-06A.Scaf21: 7251..7790	ATCC-27768.Scaf3: 43444..43980
	AF52-21.Scaf21: 16966..17502	CM04-06A.Scaf8: 19829..20365	

C.

**Gene/Protein related to Polyamines**	**AF52-21^{T}**	**CM04-06^{T}**	**ATCC 27768^{T}**
5'-methylthioadenosine nucleosidase (EC 3.2.2.16) @ S-adenosylhomocysteine nucleosidase (EC 3.2.2.9)	AF52-21.Scaf8: 69720..69037	CM04-06A.Scaf2: 169647..170330	ATCC-27768.Scaf8: 37153..36470
ABC transporter, periplasmic spermidine putrescine-binding protein PotD (TC 3.A.1.11.1)	AF52-21.Scaf1: 195853..197085	CM04-06A.Scaf1: 508153..506921	ATCC-27768.Scaf5: 149710..150942
Agmatinase (EC 3.5.3.11)	AF52-21.Scaf4: 171993..172871	CM04-06A.Scaf1: 735184..736062	ATCC-27768.Scaf4: 244627..243752

Arginine decarboxylase (EC 4.1.1.19)	AF52-21.Scaf4: 169656..171125	CM04-06A.Scaf1: 732847..734316	ATCC-27768.Scaf4: 246963..245497
Arginine/ornithine antiporter ArcD	AF52-21.Scaf13: 32456..33040	CM04-06A.Scaf16: 21346..20033	ATCC-27768.Scaf1: 228824..228243
	AF52-21.Scaf2: 163084..164484	CM04-06A.Scaf17: 39776..39192	
		CM04-06A.Scaf24: 23217..22498	
Carboxynorspermidine decarboxylase, putative (EC 4.1.1.-)	AF52-21.Scaf4: 174169..175383	CM04-06A.Scaf1: 737354..738568	ATCC-27768.Scaf4: 242455..241244
Carboxynorspermidine dehydrogenase, putative (EC 1.1.1.-)	AF52-21.Scaf4: 172910..174169	CM04-06A.Scaf1: 736095..737354	ATCC-27768.Scaf4: 243714..242455
Putrescine transport ATP-binding protein PotA (TC 3.A.1.11.1)	AF52-21.Scaf1: 193140..194186	CM04-06A.Scaf1: 510866..509820	ATCC-27768.Scaf5: 146997..148043
Spermidine Putrescine ABC transporter permease component PotB (TC 3.A.1.11.1)	AF52-21.Scaf1: 194193..195008	CM04-06A.Scaf1: 509813..508998	ATCC-27768.Scaf5: 148050..148865
Spermidine Putrescine ABC transporter permease component potC (TC..3.A.1.11.1)	AF52-21.Scaf1: 195008..195856	CM04-06A.Scaf1: 508998..508150	ATCC-27768.Scaf5: 148865..149713
Spermidine synthase (EC 2.5.1.16)	AF52-21.Scaf4: 171136..171996	CM04-06A.Scaf1: 734327..735187	ATCC-27768.Scaf4: 245484..244627

D.

**Gene/Protein related to Teichoic and lipoteichoic acids**	**AF52-21^T**	**CM04-06^T**	**ATCC 27768^T**
Cell wall teichoic acid glycosylation protein gtcA	AF52-21.Scaf5: 61372..60722	CM04-06A.Scaf5: 69650..70273	ATCC-27768.Scaf3: 73368..72748
Teichoic acid export ATP-binding protein TagH (EC 3.6.3.40)	AF52-21.Scaf12: 38837..39580	CM04-06A.Scaf15: 8981..9724	ATCC-27768.Scaf3: 196245..195502
Membrane protein involved in the export of O-antigen, teichoic acid lipoteichoic acids	AF52-21.Scaf14: 88192..89649		ATCC-27768.Scaf5: 131794..133056

E.

Gene/Protein related to Quinones	AF52-21^T	CM04-06^T	ATCC 27768^T
2-heptaprenyl-1,4-naphthoquinone methyltransferase (EC 2.1.1.163)	AF52-21.Scaf14: 50208..50891	CM04-06A.Scaf1: 760392..759709	
Electron transport complex protein RnfA	AF52-21.Scaf12: 46092..46724	CM04-06A.Scaf15: 16243..16875	ATCC-27768.Scaf3: 188001..187369
	AF52-21.Scaf8: 80810..81424	CM04-06A.Scaf2: 157530..156919	ATCC-27768.Scaf8: 43730..44341
Electron transport complex protein RnfB	AF52-21.Scaf12: 46741..47646	CM04-06A.Scaf15: 16891..17787	ATCC-27768.Scaf3: 187353..186454
Electron transport complex protein RnfC	AF52-21.Scaf12: 42672..43913	CM04-06A.Scaf15: 12850..14061	ATCC-27768.Scaf3: 191521..190196
Electron transport complex protein RnfD	AF52-21.Scaf12: 43913..44866	CM04-06A.Scaf15: 14061..15014	ATCC-27768.Scaf3: 190196..189237
	AF52-21.Scaf8: 79067..80095	CM04-06A.Scaf2: 159282..158245	ATCC-27768.Scaf8: 41992..43011
Electron transport complex protein RnfE	AF52-21.Scaf12: 45425..46087	CM04-06A.Scaf15: 15576..16238	ATCC-27768.Scaf3: 188668..188003
	AF52-21.Scaf8: 80092..80820	CM04-06A.Scaf2: 158248..157523	ATCC-27768.Scaf8: 43008..43733
Electron transport complex protein RnfG	AF52-21.Scaf12: 44863..45408	CM04-06A.Scaf15: 15011..15559	ATCC-27768.Scaf3: 189240..188686
F420H2:quinone oxidoreductase	AF52-21.Scaf20: 16316..17497		
Heptaprenyl diphosphate synthase component I (EC 2.5.1.30)	AF52-21.Scaf1: 223454..223993	CM04-06A.Scaf1: 381659..382198	ATCC-27768.Scaf4: 12303..11758
Microsomal dipeptidase (EC 3.4.13.19)	AF52-21.Scaf2: 5281..6282	CM04-06A.Scaf10: 5031..6032	ATCC-27768.Scaf10: 50915..49926
Octaprenyl diphosphate synthase (EC 2.5.1.90) / Dimethylallyltransferase (EC 2.5.1.1) / (2E,6E)-farnesyl diphosphate synthase (EC 2.5.1.10) / Geranylgeranyl pyrophosphate synthetase (EC 2.5.1.29)		CM04-06A.Scaf1: 473857..474747	ATCC-27768.Scaf2: 124174..123293
Ubiquinone/menaquinone biosynthesis methyltransferase UbiE (EC 2.1.1.-) @ 2-heptaprenyl-1,4-naphthoquinone methyltransferase MenG (EC 2.1.1.163)		CM04-06A.Scaf10: 17887..18516	ATCC-27768.Scaf7: 119329..119943
		CM04-06A.Scaf10: 17887..18516	ATCC-27768.Scaf7: 119329..119943
Undecaprenyl diphosphate synthase (EC 2.5.1.31)	AF52-21.Scaf4: 65950..65237	CM04-06A.Scaf1: 292073..292786	ATCC-27768.Scaf4: 46136..45423

Supplementary Figure S1. Polar lipids of strains AF52-21^T, CM04-06^T, and ATCC 27768^T separated by two-dimensional TLC. Detected with (a) molybdatophosphoric acid reagent, (b) molybdenum blue reagent, (c) ninhydrin reagent, (d) anisaldehyde reagent. DPG, diphosphatidylglycerol; PG, phosphatidylglycerol; GL, unidentified glycolipid; PGL, unidentified phosphoglycolipids; PL, unidentified phospholipid; L, unidentified lipid.

AF52-21^T

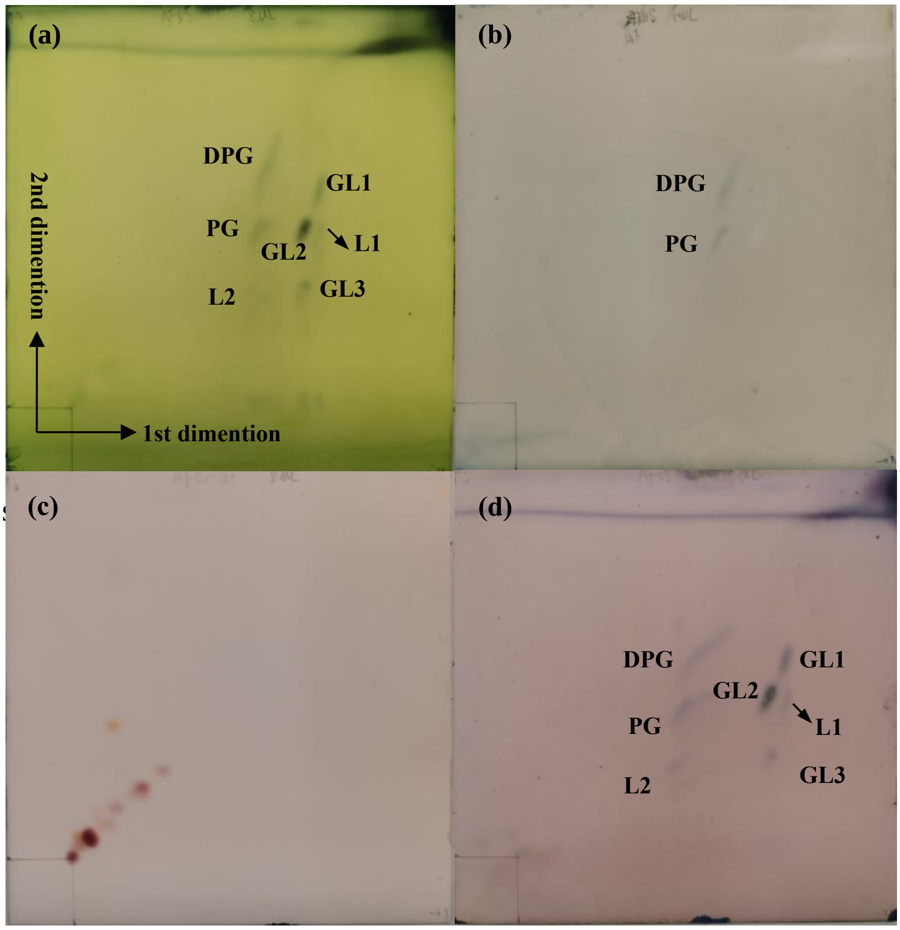

CM04-06^T

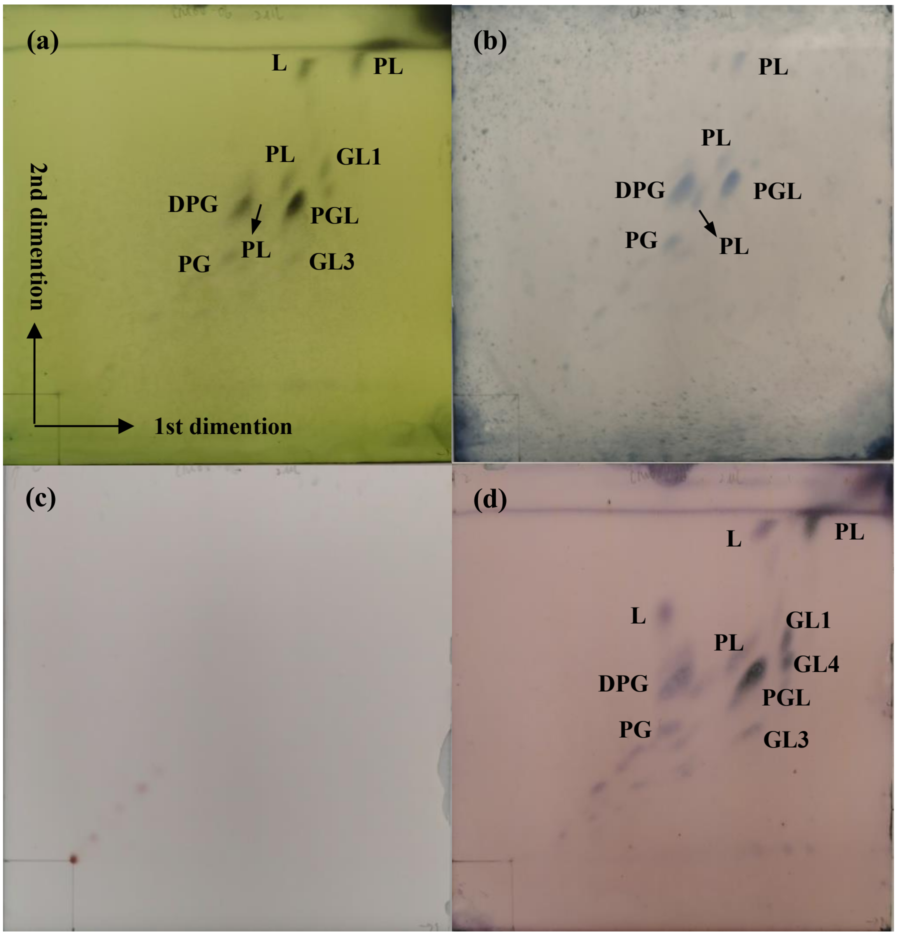

(a)
L
PL
PL
GL1
DPG
PGL
PL
PG
GL3
2nd dimention
1st dimention
(b)
PL
PL
DPG
PGL
PG
PL
(c)
(d)
L
PL
L
GL1
PL
GL4
DPG
PGL
PG
GL3

F. prausnitzii ATCC 27768^T

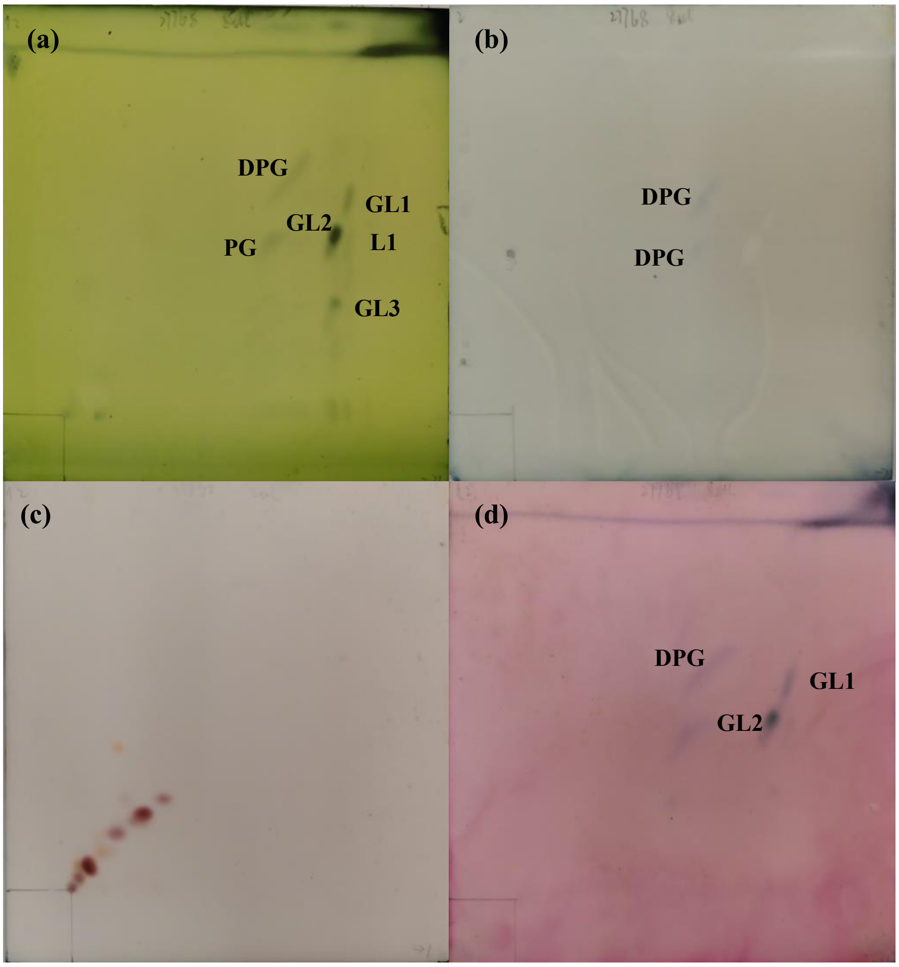

Supplementary Figure S2. Neighbour-joining phylogenetic tree based on 16S rRNA gene sequences showing the phylogenetic relationships of strains AF52-21^T, CM04-06^T and the representatives of several other related taxa. *Clostridium butyricum* DSM 10702^T (AQQF01000149) was used as an out-group. Bootstrap values based on 1,000 replications higher than 70% are shown at the branching points. Bar, substitutions per nucleotide position.

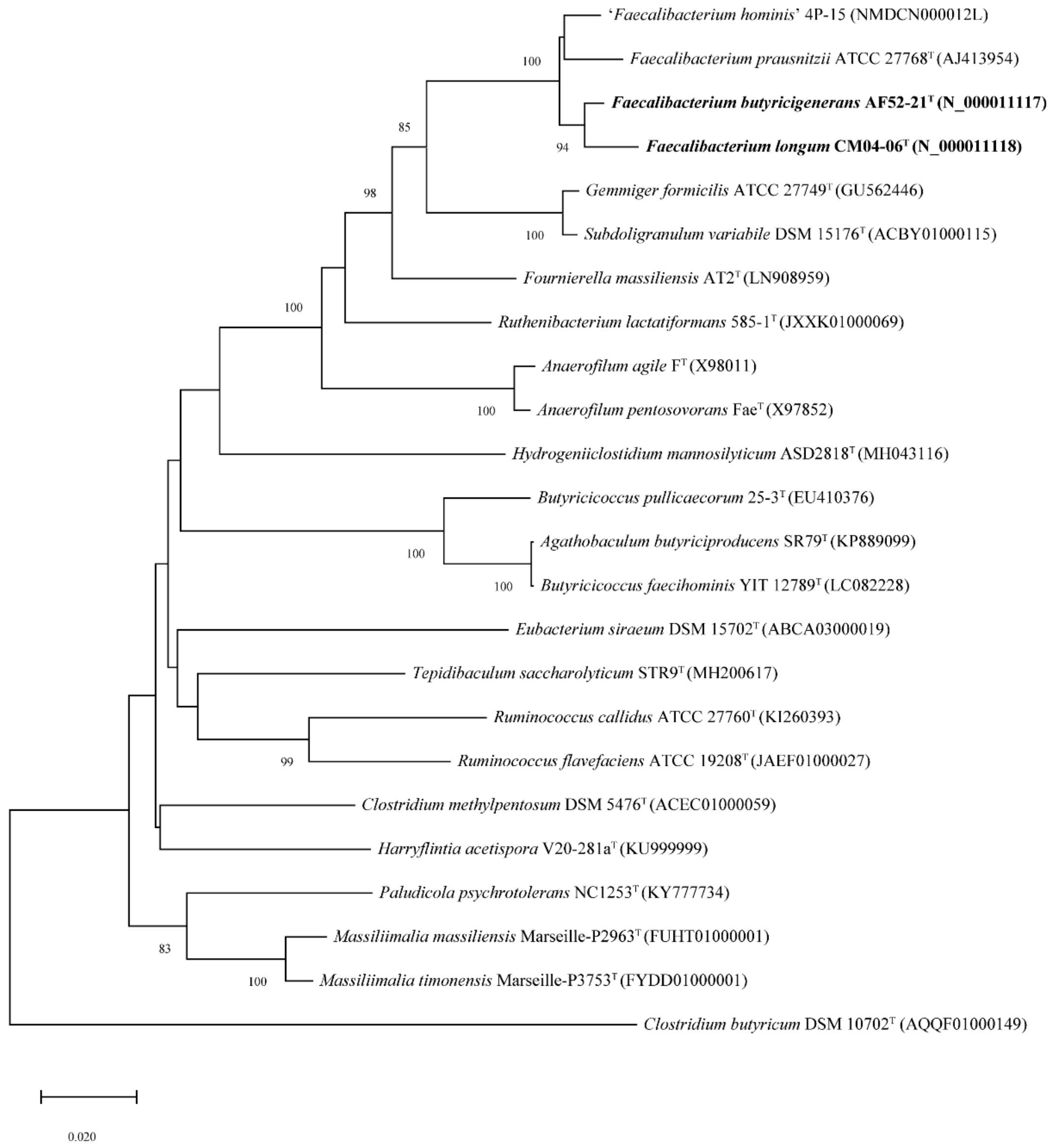

Supplementary Figure S3. Minimum-evolution phylogenetic tree based on 16S rRNA gene sequences showing the phylogenetic relationships of strains AF52-21^{T}, CM04-06^{T} and the representatives of related taxa. *Clostridium butyricum* DSM 10702^{T} (AQQF01000149) was used as an out-group. Bootstrap values based on 1000 replications higher than 70% are shown at the branching points. Bar, substitutions per nucleotide position.

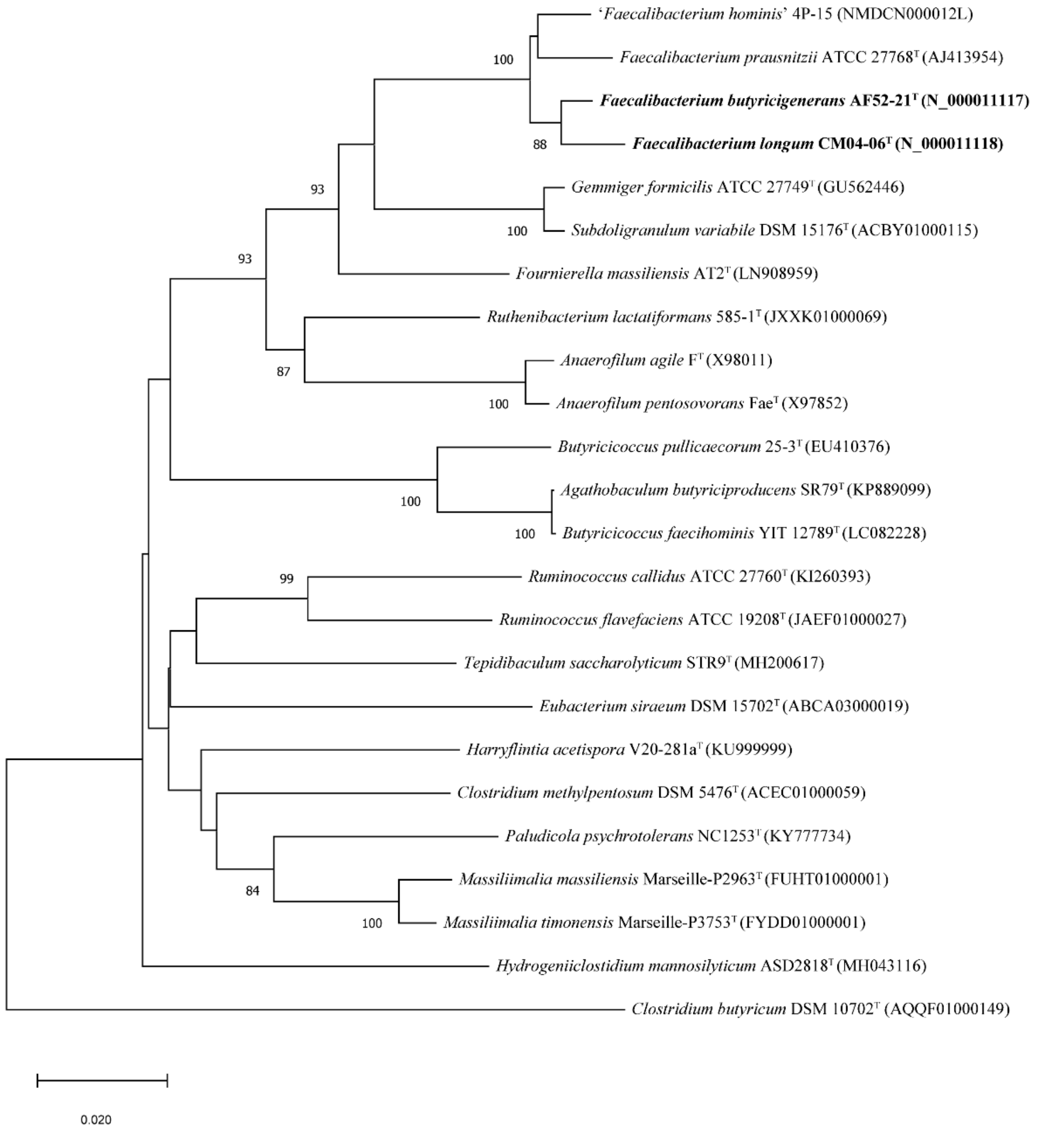

Supplementary Figure S4. Comparison of COG functional categories of strains AF52-21^{T}, CM04-06^{T} and the related species *F. prausnitzii* ATCC 27768^{T}.

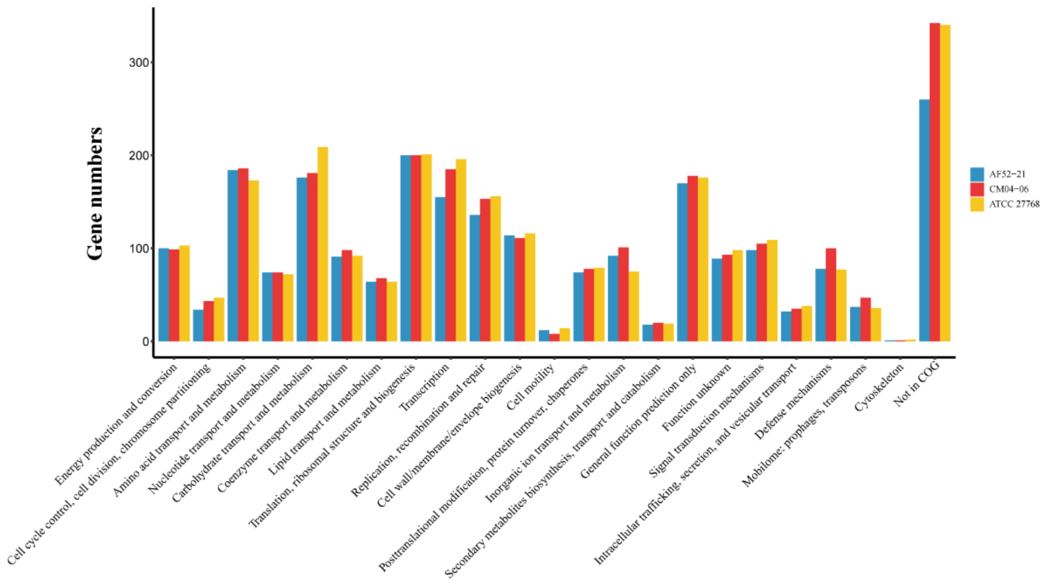

Supplementary Figure S5. Comparison of BGCs in strains AF52-21^T, CM04-06^T, and *F. prausnitzii* ATCC 27768^T.

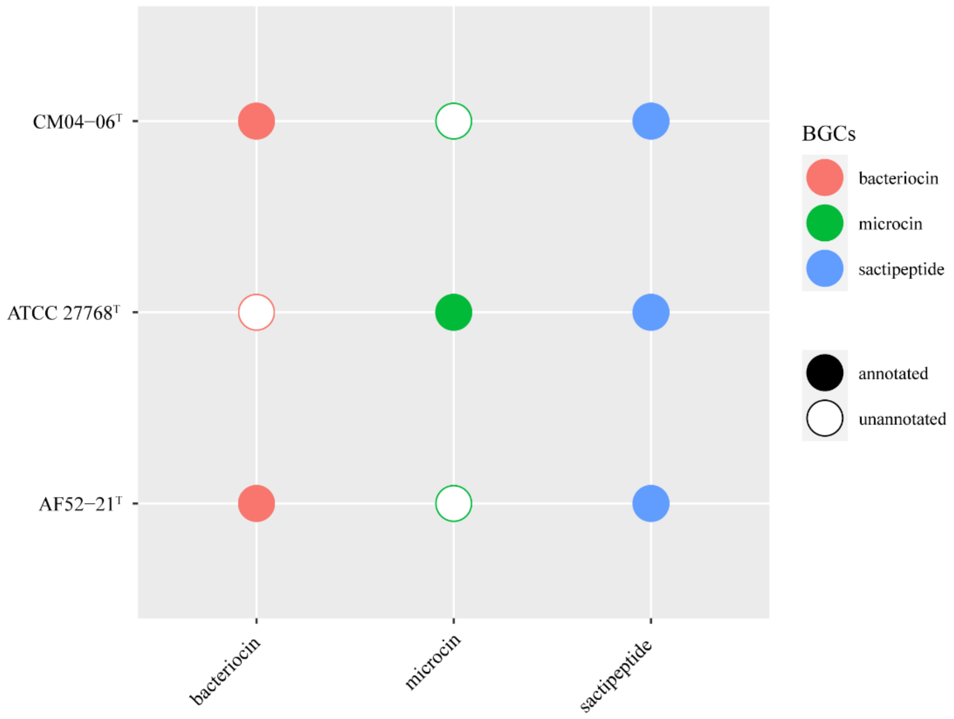

Supplementary Figure S6. Distribution of prophage in strains AF52-21^T and CM04-06^T.

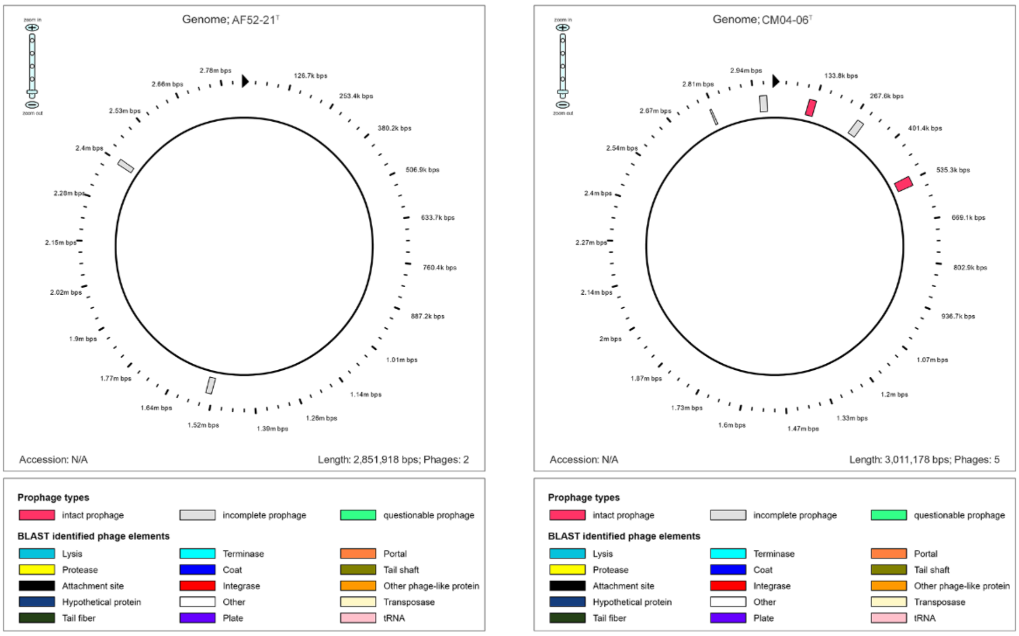

Supplement: Supplementary file 1 — Supplementary Information. [file 41598_2021_90786_MOESM1_ESM.pdf]
